# The swallow tail sign: mechanisms, neuroimaging advances and future perspectives

**DOI:** 10.1186/s13244-026-02401-6

**Published:** 2026-09-30

**Authors:** Zhixuan Li, Haoxuan Lu, Yongqin Xiong, Caohui Duan, Xin Lou

**Affiliations:** Department of Radiology, Chinese PLA General Hospital, Beijing, China

**Keywords:** Parkinson disease, Substantia nigra, Magnetic resonance imaging, Pars compacta, Biomarkers

## Abstract

**Abstract:**

Parkinson’s disease (PD), the second most common neurodegenerative disorder, poses a significant challenge to elderly health. Its diagnosis currently relies primarily on clinical symptoms, which often proves inadequate for differentiating PD from other forms of parkinsonism, particularly in early disease stages. Although positron emission tomography (PET) has offered compelling evidence supporting the differential diagnosis of PD, its high cost and radiation exposure limit its widespread use. Consequently, there is a pressing need for more accessible, convenient, non-invasive, and safe diagnostic approaches. The “swallow tail sign” (STS)—an MRI-based biomarker characterized by a hyperintensity in the posterolateral substantia nigra pars compacta (SNc), surrounded by hypointense areas—has emerged as a highly promising adjunct tool. This biomarker holds potential not only to enhance diagnostic certainty in distinguishing PD from similar neurodegenerative disorders but also to improve the understanding of its pathophysiology. In this review, we outline the anatomical basis of the STS and the pathological changes in PD leading to its disappearance. We further summarize its applications across various magnetic resonance imaging (MRI) techniques and evaluate its value in identifying PD and differentiating it from other parkinsonian syndromes. With significant advancements in ultra-high-field MRI (≥ 7 T) and artificial intelligence (AI), combined with other MRI biomarkers, the value of STS will be further explored in the future.

**Key Points:**

**Question** Despite its diagnostic potential, the biological basis, imaging interpretation, and clinical utility of the STS remain incompletely understood and warrant further investigation in parkinsonism.

**Findings** The normal STS appears as dorsolateral nigral hyperintensity on susceptibility-sensitive MRI, while its attenuation or loss in PD supports cautious adjunctive diagnostic use.

**Critical Relevance Statement** This review critically evaluates the biological basis, MRI interpretation, diagnostic limitations, and evolving multimodal and AI-based assessment of the STS, guiding radiologists toward its standardized and cautious adjunct use in the clinical imaging assessment of parkinsonism.

**Graphical Abstract:**

**Figure:**
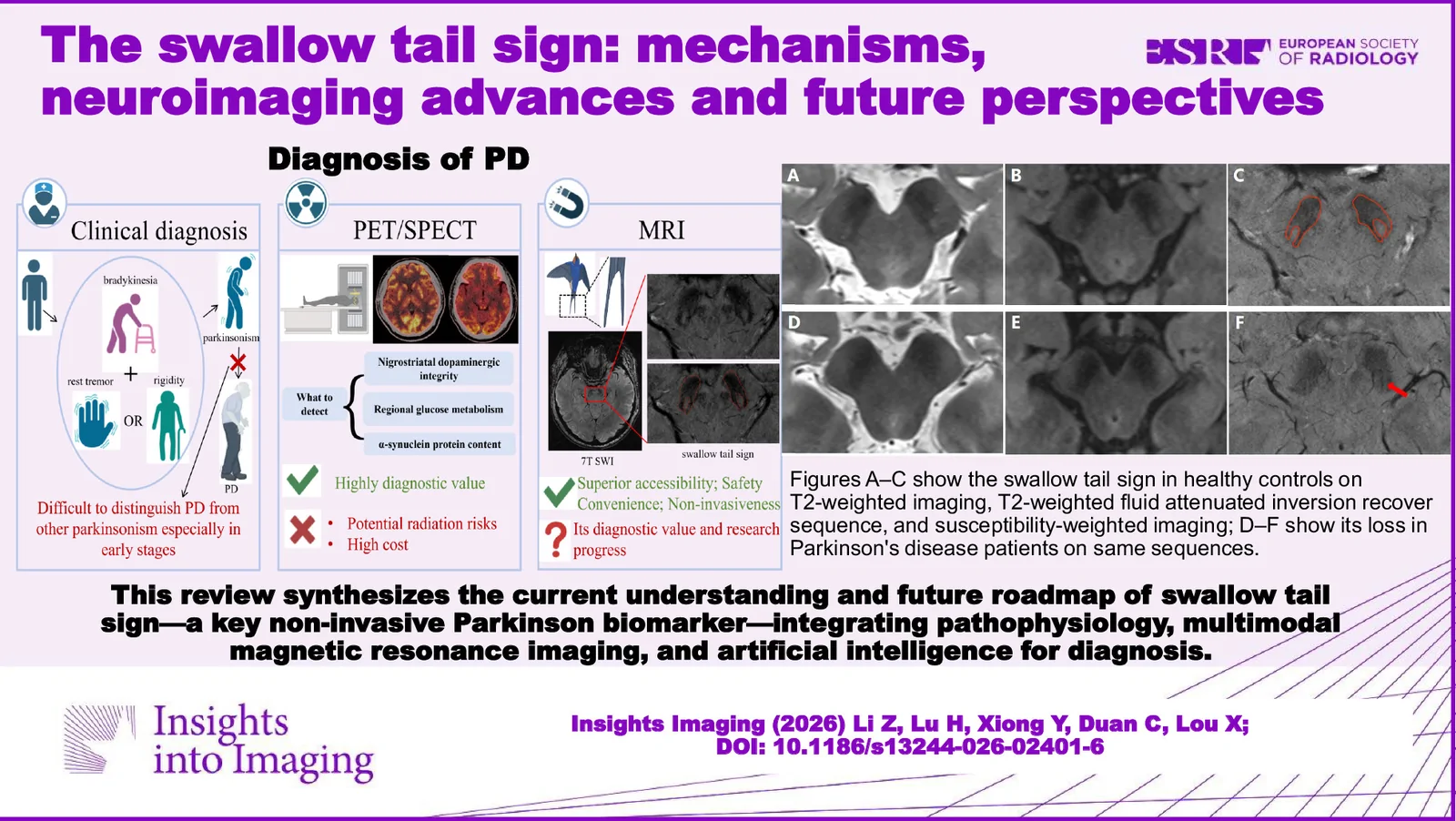

## Introduction

Parkinson’s disease (PD), the second most common neurodegenerative disorder, is characterized clinically by bradykinesia, rigidity, rest tremor, and postural instability, while its key pathological feature is the degeneration and loss of dopaminergic neurons in the substantia nigra (SN) [1, 2]. Over the past generation, largely driven by population aging, the global burden of PD has more than doubled, with current estimates suggesting that approximately 11.8 million individuals are affected worldwide [3]. Currently, the diagnosis of PD relies primarily on clinical evaluation. According to the Movement Disorder Society clinical diagnostic criteria, the diagnostic process first requires the confirmation of parkinsonism, defined by bradykinesia plus one of resting tremor and rigidity, or both, and then applying some specific criteria to determine if the syndrome is attributable to PD [4]. However, from a clinical perspective, using these specific criteria to accurately distinguish PD from other conditions that cause similar symptoms remains a major challenge, especially in the early stages. The differential diagnosis relies heavily on the clinician’s experience to exclude other conditions, such as atypical parkinsonian syndromes (AP) (e.g., multiple system atrophy (MSA), progressive supranuclear palsy (PSP), corticobasal degeneration (CBD)), vascular parkinsonism, Lewy body dementia, and essential tremor (ET).

**Fig. 1:**
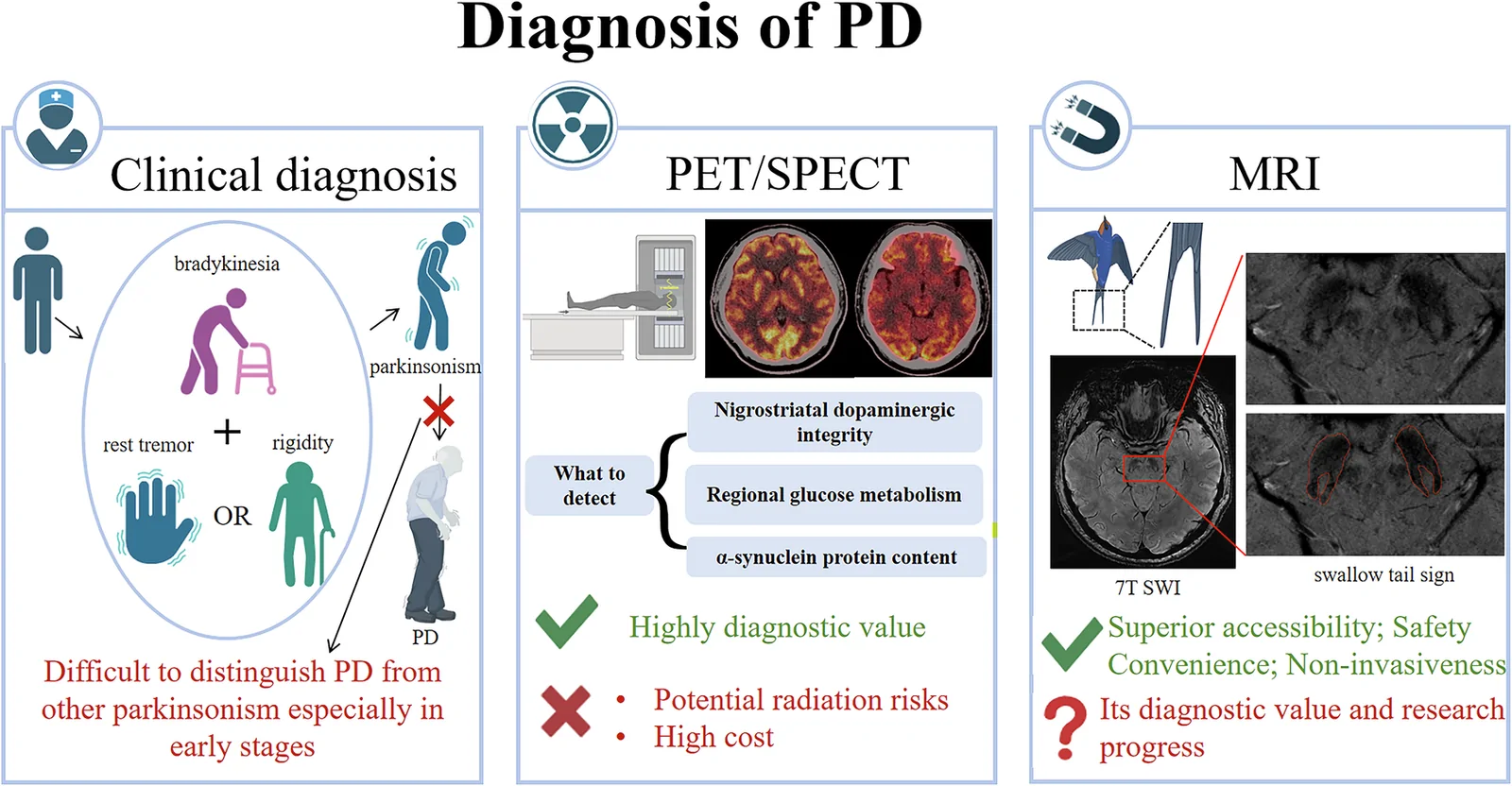
The three main diagnostic methods of PD

Recent advances in positron emission tomography (PET) and single-photon emission computed tomography (SPECT) have provided substantial evidence to support the diagnosis of PD and its differentiation from other parkinsonian disorders. Several functional imaging techniques serve as valuable tools for evaluating nigrostriatal dopaminergic integrity, including dopamine transporter (DAT), vesicular monoamine transporter (VMAT), and iodine123-2β-carbomethoxy-3β-(4-iodophenyl)-*N*-(3-fluoropropyl)-nortropane (^123^I-FP-CIT) SPECT [5–7]. Additionally, ^18^F-FDG PET technology clearly reveals the disease-specific patterns of regional glucose metabolism [8, 9]. Novel radiotracers such as ^18^F-SPAL-T-06 and ^18^F-F0502B, which specifically target the α-synuclein protein, an important pathological hallmark of PD within the nigrostriatal pathway, have shown promising potential [10, 11]. These imaging modalities help support the diagnosis of PD and facilitate its differentiation from other parkinsonian disorders. However, the potential radiation risks associated with injecting radiotracers and the high cost of nuclear medicine imaging limit their widespread clinical use. Moreover, extensive research indicates that it lacks sufficient reliability to differentiate between PD and APs or to rule out alternative diagnoses, such as cerebrovascular disease [12]. In contrast, magnetic resonance imaging (MRI) is more accessible, convenient, non-invasive, and safe, making MRI biomarkers for PD an increasingly compelling research focus. The three main diagnostic approaches for PD are illustrated in Fig. 1.

In 2014, Schwarz et al first described the “swallow tail sign” (STS) on standard axial high-resolution 3-T susceptibility-weighted imaging (SWI). This sign is characterized by a hyperintensity in the posterolateral one-third of the substantia nigra pars compacta (SNc), which is surrounded by relatively hypointense areas, creating an appearance reminiscent of a swallow’s tail. They proposed that its absence could serve as a triage marker supporting the distinction between degenerative parkinsonism and non-degenerative mimics. Furthermore, within workflows aimed at distinguishing PD from AP, it could act as a valuable adjunct—particularly in cases where STS alone may be insufficient—as part of a broader multimodal imaging panel [13]. As a simple qualitative imaging biomarker for PD, the STS has subsequently become the focus of extensive and ongoing research.

Against this background, this review synthesizes current knowledge on STS, organized into several key sections: First, we outline the anatomical basis of the STS and the pathological changes in PD that lead to its loss. Subsequently, we review the existing literature to summarize the applications of STS across different MRI modalities and to evaluate its usefulness in identifying PD and distinguishing it from other parkinsonian disorders. With ongoing advancements in imaging technology, such as higher resolution and optimized parameters, as well as the significant progress in machine learning (ML), STS may play an increasingly vital role in enhancing the accuracy of PD diagnosis and differentiation from other parkinsonian syndromes.

## Materials and methods

To survey the published articles, we conducted a thorough literature search in PubMed up to November 2025, using terms including “PD”, “parkinson’s disease”, “parkinson”, “atypical parkinsonism”, “parkinsonism”, “swallow tail”, “swallow tail sign”, “N1 sign”, “nigrosome-1 sign”, “dorsolateral nigral hyperintensity”. The inclusion criteria were clinical retrospective or prospective studies focused on STS, and the exclusion criteria included reviews, meta-analyses, or other literature types without original data. Study selection was based on screening the titles and abstracts according to the prespecified criteria, and to minimize the risk of omitting potentially relevant literature, full texts were retrieved and assessed when the eligibility was unclear. After carefully reviewing the retrieved literature, a total of 52 articles were ultimately kept that were directly related to STS.

## Structural basis of the STS and its mechanism-based alterations

### Normal structural basis of the STS

SN, located in the ventral tegmentum of the midbrain, is anatomically divided into the pars reticulata (SNr) and the pars compacta. The SNr is located in the ventral part of the SN, mainly composed of GABAergic neurons, while the SNc is located in the dorsal part, mainly composed of dopaminergic neurons that are rich in neuromelanin (NM) [14].

**Fig. 2:**
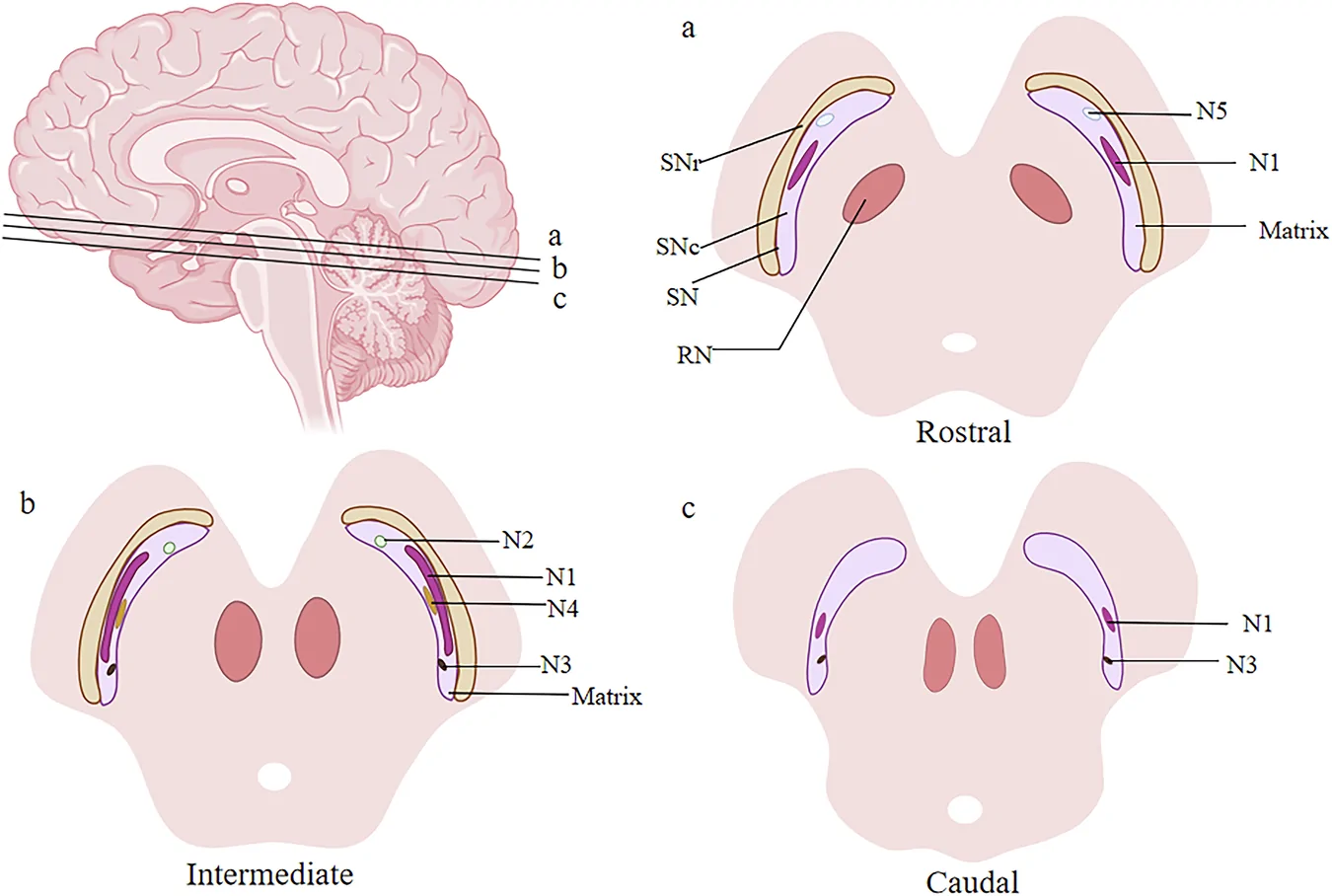
Anatomical subdivisions of the SN at three representative axial levels of the midbrain. The three cross-sections correspond to the upper midbrain (**a**), the mid-midbrain at the level where N1 is most prominent (**b**), and the lower midbrain near the midbrain–pons junction (**c**). These sections illustrate the spatial organization of the SN and its subregions across the rostro-caudal axis. SN, substantia nigra; SNc, substantia nigra pars compacta; SNr, substantia nigra pars reticulata; RN, red nucleus; N, nigrosome

In 1999, Damier et al. first described that the SNc is further subdivided into nigrosomes and the nigral matrix according to specific immunohistochemical staining characteristics [15]. The nigrosomes were mainly composed of five calcium-binding protein-negative regions containing dopaminergic neurons, designated as Nigrosome-1 (N1) to Nigrosome-5 (N5). Approximately 60% of all dopaminergic neurons in the SN are located within the nigral matrix, while the remaining 40% are distributed across the five nigrosomes. Among these, Damier et al identified N1, the largest nigrosome—situated in the ventrolateral region of the SN—as the most severely affected in PD, exhibiting a dramatic loss of up to 98% of its dopaminergic neurons. Additionally, the degree of neuronal loss is ranked in the following order: N1 > N2 > N4 > N3 > N5 [16] (Fig. 2).

In T2-weighted images (T2WI) and SWI of healthy individuals, the N1 showed hyperintensity, surrounded by the adjacent hypointense SN and medial lemniscus, forming a distinctive “swallow tail” [13]. Historically, the hyperintense inner region of STS has been conventionally attributed to N1, so the STS is also known as “N1 sign” [17]. For research purposes, it is also preferable to use the descriptive term “dorsolateral nigral hyperintensity” to describe STS [18]. However, a 2022 study by Brammerloh et al, combining 7-T in vivo MRI, 7-T postmortem MRI, three-dimensional block-face imaging, and immunohistochemistry, revealed that the STS only partially overlaps with N1, which is thinner, longer, and extends beyond the STS in both the anteroposterior and superoinferior dimensions [19]. Nevertheless, the non-equivalence between STS and N1 does not compromise the value of STS as a biomarker for PD. Therefore, for clarity and consistency, the term STS will be used uniformly throughout the remainder of this review. However, a more precise investigation into the relationship between MRI characteristics and the anatomy of the SN may pave the way for improved diagnosis and disease monitoring in PD in the future.

### Mechanisms underlying the loss of the STS in PD

When using MRI to evaluate PD, NM and brain iron content serve as important imaging biomarkers, offering valuable pathophysiological insights into the onset and progression of PD and other neurodegenerative disorders. The literature consistently supports the view that, in patients with PD, NM levels decrease and iron content increases in the SNc [20–22].

NM is an intraneuronal pigment formed through the auto-oxidation of catecholamines. It is predominantly found in specific brainstem structures such as the SNc, ventral tegmental area, and locus ceruleus. Furthermore, NM acts as a metal ion chelator capable of binding iron. As a result, in the SNc of healthy individuals, a portion of iron exists in the form of NM–Fe complexes. In PD patients, however, the chelating function of NM is impaired, leading to the release of iron from these complexes and a consequent increase in free iron levels [23]. The loss of dopaminergic neurons in SNc causes NM to enter a depigmented state, which is thought to facilitate the deposition of its previously bound iron and other metals into adjacent brain tissues [24]. The released free iron, which is highly reactive, generates free radicals in the surrounding region via the Fenton reaction, promoting oxidative stress and other related reactions. These processes, in turn, contribute to the degeneration and loss of dopaminergic neurons in the SN, and the progressive depletion of these neurons further drives the pathogenesis and progression of the disease. Moreover, dysregulated levels of ferrous iron can drive neuroinflammation through various neurodegenerative processes [23].

**Fig. 3:**
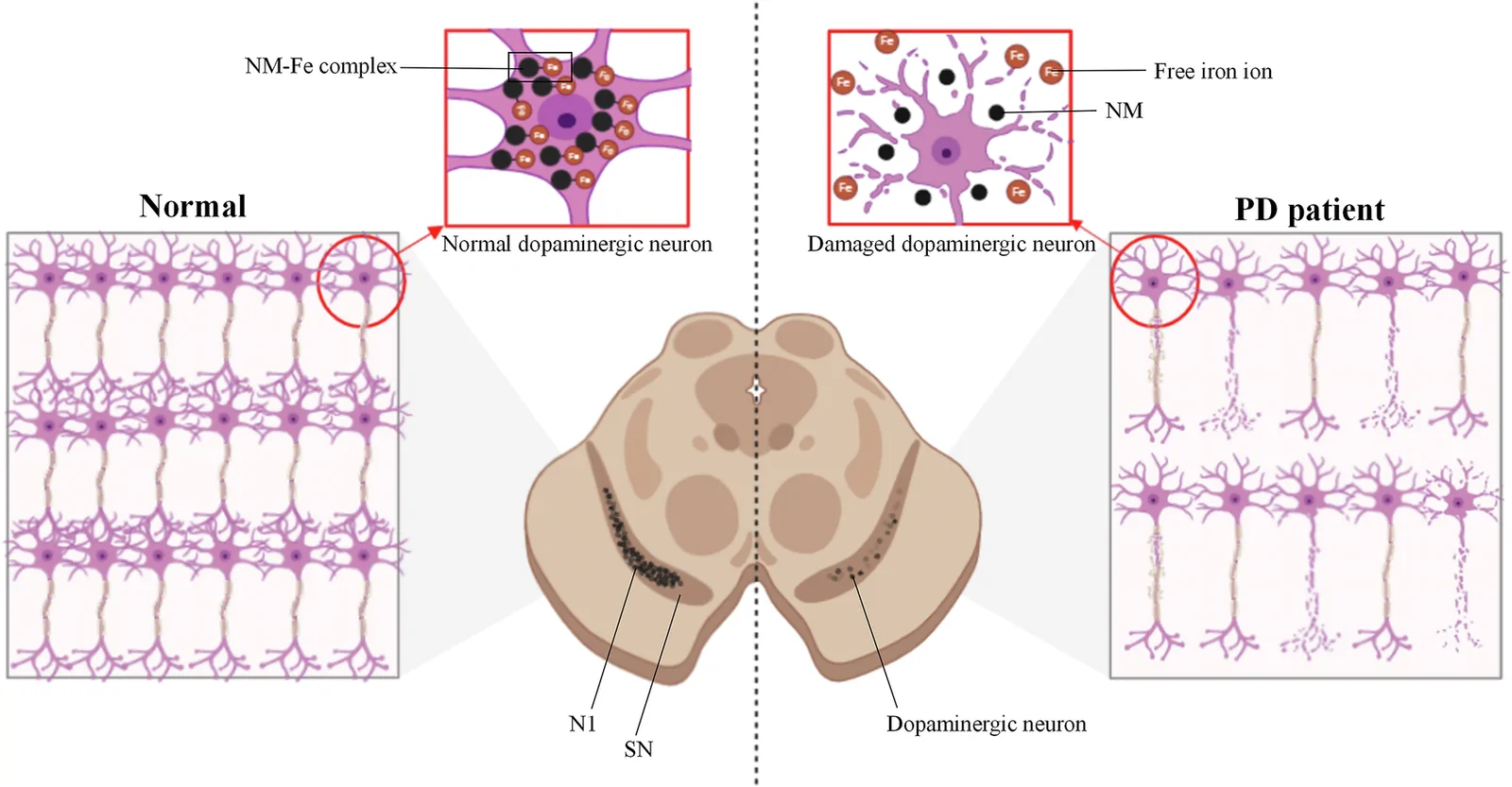
Comparison of dopaminergic neurons, NM, and iron in N1 of SN between normal individuals and PD patients. Left (Normal): In the N1 region, dopaminergic neurons are intact. NM and iron exist as stable NM-Fe complexes within the cytoplasm. Right (PD patient): in the N1 region, the number of dopaminergic neurons is reduced, and some neurons are disintegrated. The NM-Fe complexes are disrupted, releasing free NM and free iron ions

Taken together, the disappearance of the STS on MRI mainly results from the following mechanism: the progressive loss of dopaminergic neurons in N1 region leads to reduced NM content and an accumulation of paramagnetic free iron (Fig. 3). These changes may cause a decrease in signal intensity on T2WI or SWI, making the region isointense with the surrounding structures and thereby resulting in the apparent “loss of STS” on MRI.

### Possible causes underlying the loss of the STS in healthy people

**Fig. 4:**
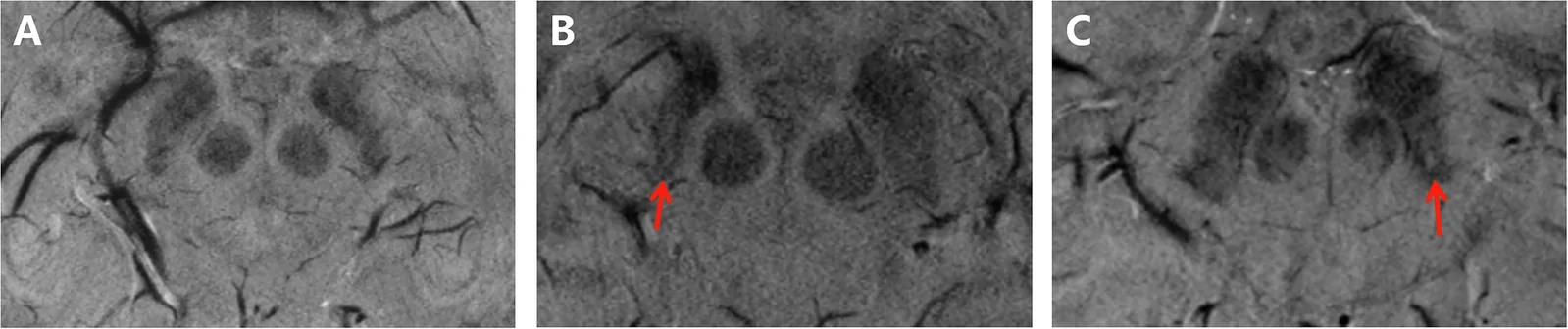
Variable visualization of the STS in HCs on 7-T SWI. The three panels in this figure show SWI images from three HCs acquired on a 7-T MRI system (MAGNETOM Terra, Siemens Healthcare). **A** Image from a 66-year-old healthy female subject. Bilateral STS is poorly visualized. **B** Image from a 71-year-old healthy male subject. The right-sided STS is possibly visualized (red arrow), and the left-sided STS is not clearly identified. **C** Image from a 51-year-old healthy female subject. A subtle hyperintense signal is observed in the left SN region (red arrow), whereas the right-sided STS is not clearly visualized. All images were obtained from the Department of Radiology, First Medical Center of Chinese PLA General Hospital

However, the absence of the STS is not unique to patients, as it can also occur in the healthy controls (HCs), as illustrated in Fig. 4. First, a postmortem 7-T SWI-MRI study revealed that the disappearance of STS is attributable not only to the iron deposition in brain parenchyma but also to the presence of microvessels containing iron or deoxyhemoglobin. At ultra-high field strengths, both iron deposition and microvascular structures collectively contribute to the hypointensity in the N1 region and its surroundings. Such microvascular features within the midbrain dopaminergic system may lead to false-positive interpretations of STS changes [25]. Second, studies have shown that approximately 19% of HCs exhibit an absence of STS, likely due to individual anatomical variations or physiological iron deposition associated with normal aging [26, 27]. Third, inter-individual differences in the content and molecular composition of NM within the SNc may lead to incomplete hyperintense visualization of STS, resulting in its absence in some HCs [28]. Moreover, since STS evaluation relies on MRI, false positives can also arise from technical factors such as the scanning equipment, sequence parameters, the amplification effects of iron-sensitive sequences, radiologists’ expertise, and motion artifacts.

## Recent progress in STS imaging

### Iron-sensitive susceptibility imaging

#### SWI

The appearance and disappearance of STS is closely related to the level of iron content within the N1 region, making iron-sensitive susceptibility imaging a key radiological technique for its evaluation. Although conventional T2WI can reflect iron deposition, its sensitivity is limited [29]. Studies have indicated that SWI is currently a highly sensitive MRI sequence for assessing iron deposition in neurodegenerative diseases [30, 31]. SWI exploits magnetic susceptibility differences between tissues and is particularly sensitive to substances that induce local magnetic field inhomogeneities, such as iron and calcium. Its technical principles include the use of long echo times, three-dimensional gradient echo acquisition, and post-processing of phase information, which together enable the generation of high-contrast images that significantly enhance the detection of susceptibility-sensitive substances and distinguish between paramagnetic (e.g., iron) and diamagnetic (e.g., calcium) materials. Evolving from simple two-dimensional T2*-weighted sequences to three-dimensional sequences, SWI has improved spatial resolution and enhanced sensitivity and contrast [29, 31].

**Fig. 5:**
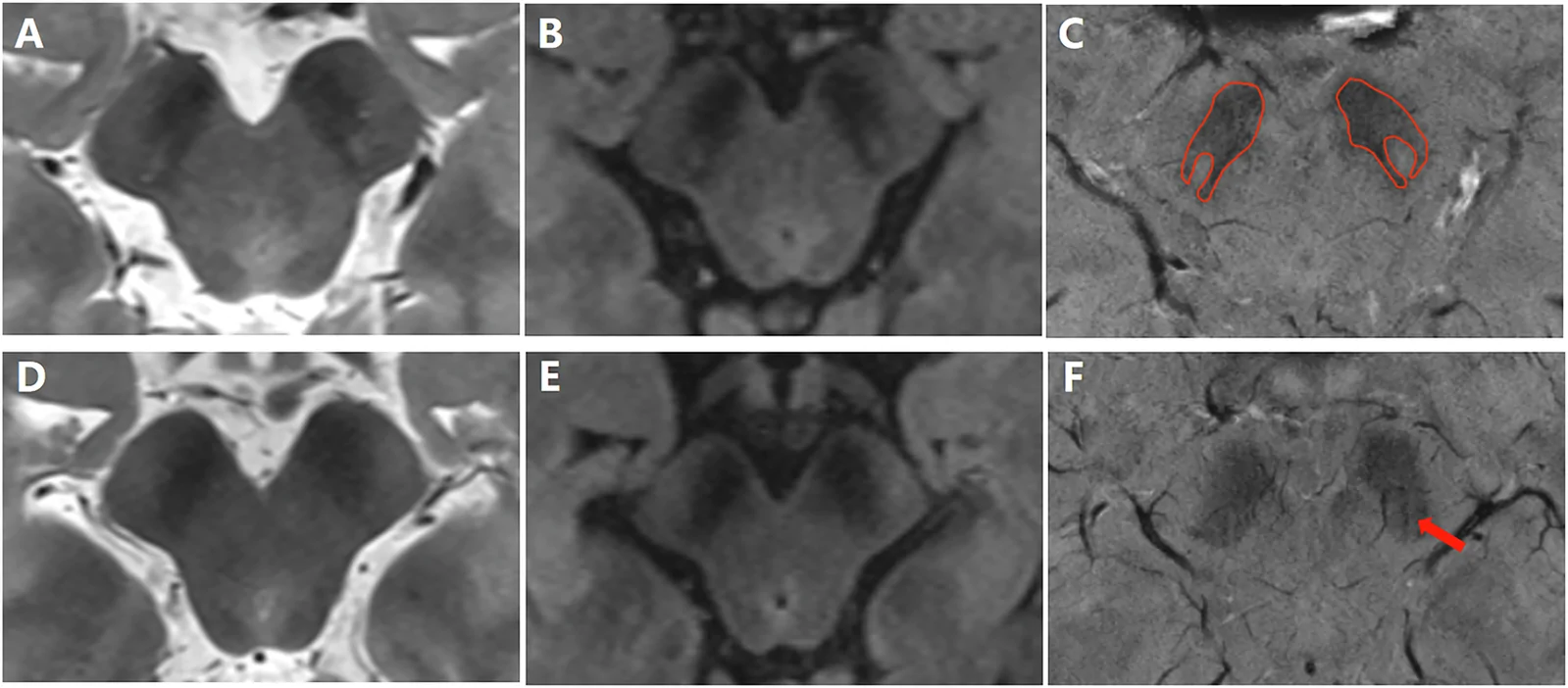
**A**–**C** T2WI, T2-FLAIR, and SWI images from a 54-year-old healthy female subject, acquired on the same scanner (7-T, MAGNETOM Terra, Siemens Healthcare) at the same slice level. The STS is visible in all sequences, with SWI providing the most distinct visualization. The area outlined by the red line in (**C**) shows the STS. **D**–**F** T2WI, T2-FLAIR, and SWI images from a 67-year-old female patient with PD, acquired on the same scanner (7-T, MAGNETOM Terra, Siemens Healthcare) at the same slice level. The STS is absent on T2WI and FLAIR images. On SWI, the STS is absent on the right side, while a slightly hyperintense signal is observed in the left SN (red arrow). All the images in this figure were obtained from the Department of Radiology, First Medical Center of Chinese PLA General Hospital

The STS can be visualized on both T2WI [32] and T2-weighted fluid-attenuated inversion recovery sequences (T2-FLAIR) [33]. However, compared with T2WI and T2-FLAIR, SWI offers a higher contrast-to-noise ratio (CNR) and a clearer depiction of STS anatomy, enabling more distinct visualization of structures such as N1 [29, 34] (Fig. 5). Compared to single-echo SWI, multi-echo SWI can further improve the CNR, attributed to increased signal-to-noise ratio (SNR). Moreover, studies indicate that there is no significant difference in CNR measurements between 20-channel and 64-channel coils for visualizing anatomical details of STS, suggesting that SWI performance in STS visualization may not be affected by the number of coil channels [34]. However, slice thickness is critical for diagnostic accuracy. One study indicated that when using a clinical 3-T SWI sequence with a slice thickness of 3 mm, STS had limited diagnostic value in distinguishing PD from AP, likely due to partial volume effects from excessive slice thickness, which hindered accurate identification of the N1 [35]. When the slice thickness was reduced from 2 mm to 1.5–1.6 mm, accuracy improved from 67% to 84%. Additionally, the appearance of the STS varies depending on the scanner supplier and protocol, necessitating standardized evaluation procedures [36].

However, the use of SWI to assess the STS for PD diagnosis remains controversial. The study by Sotoudeh et al reported that visual assessment of STS absence on SWI demonstrated extremely high diagnostic value for PD, with reported sensitivity of approximately 100% and specificity of about 95% [30]. A recent study by Xing et al showed that in clinically uncertain parkinsonian syndromes, evaluating the absence of STS using high-quality 3-T SWI yielded excellent diagnostic performance, which shows 91% sensitivity, 87% specificity, 90% accuracy, and a positive predictive value as high as 95%. Its diagnostic value was comparable to the current gold standard, ^123^I-FP-CIT, suggesting its potential as an effective clinical triage tool [37]. A study shows that functional PET imaging (6-[^18^F]FDOPA) still holds a diagnostic advantage over STS evaluation [38]. A systematic review found that the absence of STS had high diagnostic accuracy in distinguishing patients with parkinsonism from those with non-degenerative movement disorders, with an overall sensitivity ranging from 73% to 100% and specificity from 75% to 100% [39]. Additionally, a meta-analysis indicated that in non-degenerative parkinsonian disorders, such as vascular or drug-induced parkinsonism, the absence of STS was observed in only 21.7% of cases, suggesting that the absence of STS is more likely to indicate a neurodegenerative etiology [40]. Furthermore, the absence of STS shows potential in identifying individuals with rapid eye movement sleep behavior disorder (RBD) who are at high risk of conversion to PD [39, 41, 42].

Nevertheless, Prasuhn et al reported contrasting findings, observing no statistically significant difference in STS absence between PD and HC and noting low diagnostic performance (unilateral sensitivity 37%, bilateral sensitivity only 9%; unilateral specificity 78%, with an overall area under the ROC curve of only 0.5). They concluded that simple binary visual assessment of STS provides limited value for diagnosing PD in routine clinical practice [43]. Therefore, when using STS for diagnostic evaluation of PD, it is important to pay attention to the assessment method. As described by Schwarz et al the most reliable method currently recognized is the evaluation using a 6-point visibility scale for each hemi-mesencephalon [44].

Importantly, several neurological disorders may mimic STS abnormalities and limit its diagnostic specificity, which should be considered during image interpretation. Kim et al suggested that STS abnormalities alone cannot serve as a reliable biomarker for diagnosing idiopathic PD and may only indicate degeneration of N1 [45]. A meta-analysis revealed that STS disappearance occurred in 89.4% of AP, making it difficult to distinguish PD from these overlapping conditions [40]. Large-scale 3-T SWI studies have demonstrated that loss of nigral hyperintensity can be observed in PD, MSA, and PSP, reflecting overlapping abnormalities in N1 visualization and limiting its ability to differentiate parkinsonian disorders [18, 40, 46, 47]. In contrast, CBD generally preserves the nigrosome structure, although overlap may still occur in individual cases [48].

**Fig. 6:**
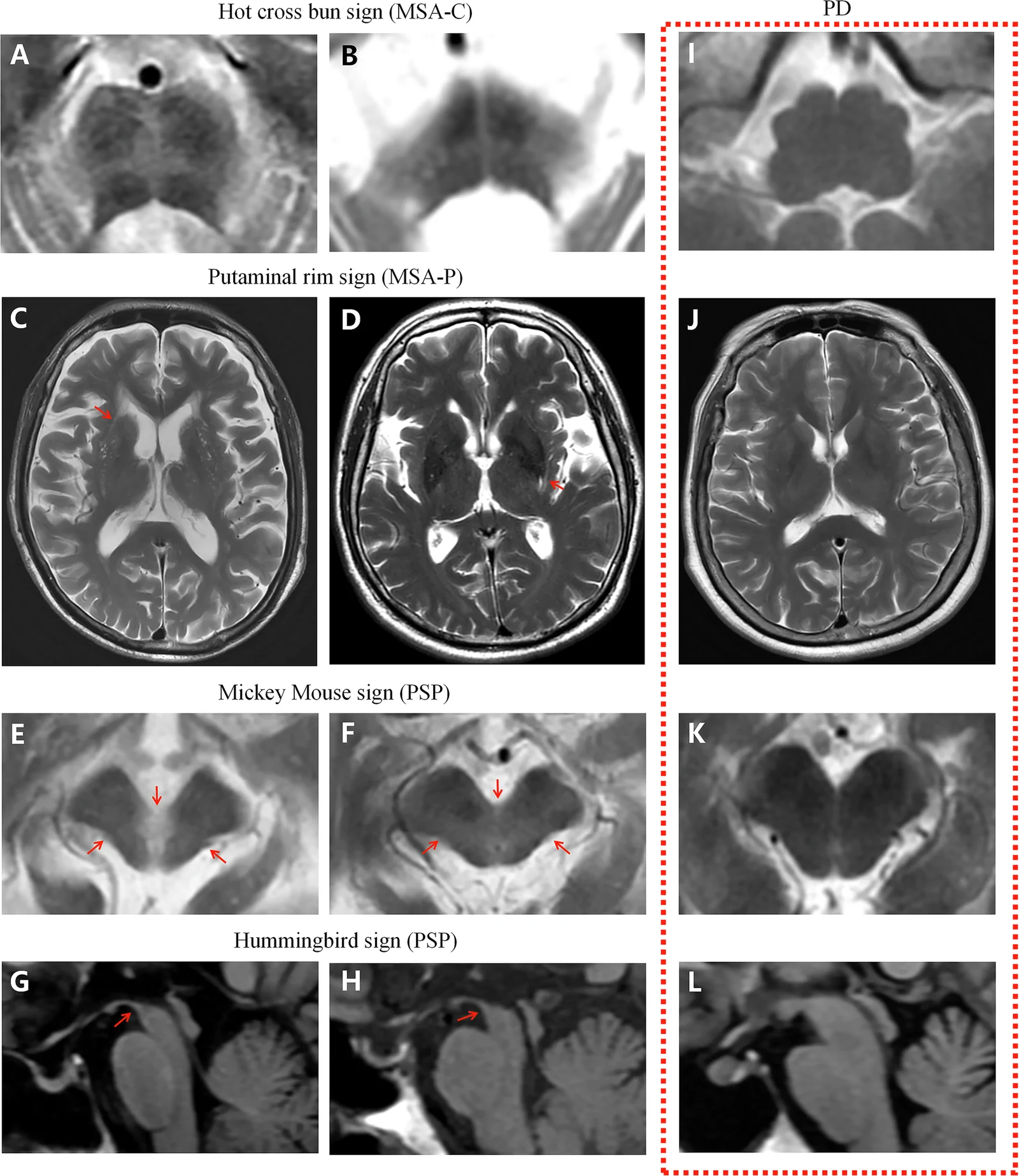
Additional MRI findings for differential diagnosis of APs. **A**, **B** Axial T2WIs from two patients with MSA-cerebellar variant (MSA-C) demonstrate pontine atrophy with cruciform hyperintense signals within the pons, corresponding to the characteristic “hot cross bun sign”. **C**, **D** Axial T2WI from two patients with MSA-parkinsonian variant (MSA-P) demonstrate a hyperintense rim along the lateral margin of the putamen (red arrows), representing the characteristic “putaminal rim sign”. **E**, **F** Axial T2WI from two patients with PSP demonstrate selective midbrain atrophy (red arrows) with relative prominence of the bilateral cerebral peduncles, resulting in the characteristic “Mickey Mouse sign”. **G**, **H** Sagittal T1-weighted images (T1WI) from two patients with PSP demonstrate marked midbrain atrophy with relative preservation of the pons, producing the characteristic “hummingbird sign” (red arrows). **I**–**L** Corresponding axial T2WI and sagittal T1WI images from patients with PD are provided for comparison. All images in this figure were obtained on a 3-T MRI scanner (Discovery 750, GE Healthcare) from the Department of Radiology, First Medical Center of Chinese PLA General Hospital

Therefore, STS should be regarded as an indicator of N1 degeneration rather than a disease-specific biomarker. Careful consideration of potential mimickers is essential in clinical interpretation. Additional MRI findings may aid in the differential diagnosis of APs. Pontine and cerebellar atrophy accompanied by the “hot cross bun sign” are characteristic imaging features of the MSA cerebellar variant (MSA-C), whereas the “putaminal rim sign” is typically observed in patients with MSA parkinsonian variant (MSA-P). In contrast, PSP is characterized by selective midbrain atrophy with the axial “Mickey Mouse sign” and sagittal “hummingbird sign” (Fig. 6) [12, 49]. When diagnostic uncertainty still remains, dopaminergic functional imaging, such as DAT-SPECT or ^18^F-DOPA PET, may provide complementary information.

Moreover, other neurodegenerative and non-neurodegenerative disorders may also present with STS abnormalities. For example, studies have reported that the absence of STS on SWI has considerable diagnostic value in distinguishing dementia with Lewy bodies from other types of dementia, with sensitivity and specificity reaching 82% and 79%, respectively [50, 51]. Furthermore, visual assessment of STS has been proposed as a potential marker of nigral neurodegeneration in α-synucleinopathies, with reported sensitivity and specificity of 85% and 82% [52]. STS absence has also been reported in multiple sclerosis [53] and even neurosyphilis [54], possibly related to iron deposition or structural alterations within the SN, further underscoring its lack of disease specificity. Non-degenerative parkinsonian disorders, including vascular parkinsonism and drug-induced parkinsonism, usually show preserved visibility of the STS, however, occasional STS abnormalities have been reported, further emphasizing that STS loss is not disease-specific [39, 45].

In addition to PD diagnosis, studies have also investigated the clinical manifestation, progression, and treatment of STS and PD. Studies have confirmed that abnormal STS is commonly observed contralateral to the affected limb [55]. However, in clinical practice, abnormal STS occasionally appears on the ipsilateral side as well. Some researchers suggest that in the context of severe neuronal loss in N1, the dopaminergic neurons in the remaining smaller clusters may take on greater significance, potentially accounting for the lack of concordance with clinical asymmetry observed in certain patients [5]. Studies have reported that in PD patients, the absence of STS is not associated with Hoehn-Yahr stage (*p* = 0.189) or with medication treatment (*p* = 0.833), indicating that STS cannot be used for “staging assessment” or “treatment monitoring”. The presence of STS is unaffected by Parkinson’s medication, suggesting that STS reflects irreversible structural pathology in the SN rather than reversible functional states [56, 57].

#### Optimized iron-sensitive imaging

Traditional SWI processing employs a mask on the high-pass filtered phase images, which can enhance the visibility of veins and iron but is geometry-dependent, potentially compromising susceptibility contrast and reducing STS recognition effectiveness [58, 59]. Furthermore, a key limitation of STS is that it is fundamentally a qualitative imaging marker, rendering its evaluation susceptible to human subjectivity and devoid of quantitative information. To address these limitations, magnetic-sensitive imaging technology has undergone significant evolution.

Quantitative susceptibility mapping (QSM) directly calculates the susceptibility values within tissue through complex post-processing algorithms, enabling precise localization and quantitative measurement of iron content [29]. Its primary advantage lies in the fact that the final quantitative results are theoretically independent of geometry shape, echo time, or field strength, providing a more objective quantitative metric [29]. It provides a complementary strategy by directly quantifying magnetic susceptibility alterations linked to iron deposition. Nevertheless, quantitative techniques often necessitate dedicated acquisition protocols, specialized post-processing workflows, and prolonged analysis times, which may hinder their routine clinical adoption. Therefore, rather than replacing STS, QSM is expected to play a synergistic role. QSM reveals that in individuals with prodromal PD manifestations, progressively increasing nigral iron deposition can be detected as the disease advances [60]. Abnormalities in QSM are already present in the early stages of the disease. However, no significant further progression is observed between early and mid-to-late stages, suggesting that these changes may reach a “plateau” early in the disease course, making it a sensitive early diagnostic biomarker, but may not be an ideal indicator for tracking long-term disease progression [57].

Susceptibility map-weighted imaging (SMWI) and true susceptibility-weighted imaging (tSWI) are techniques that combine the advantages of both QSM and SWI. Specifically, they utilize QSM data—particularly based on the high-pass filtered phase data—to generate a susceptibility mask, replacing the traditional phase mask used in SWI [23]. These approaches effectively eliminate geometric dependency, allowing prominent visualization of veins (and iron deposits) independent of their orientation. Moreover, the interference from iron signals in NM-rich regions of the SNc is better avoided, leading to a clearer and more consistent visualization of STS [23].

For STS imaging, researchers have proposed specific optimization protocols. Compared to conventional SWI, SMWI provides higher CNR and reduces partial volume effects [17]. A study suggests that in the early diagnosis and screening of PD, SMWI combined with refined scoring systems could position STS as a highly promising first-line imaging biomarker capable of replacing PET, with a sensitivity of 94.59% and a specificity of 92.49% [61].

As for tSWI, it enables more reliable visualization of the STS via a stable and rapid (< 6 min) imaging protocol that adopts finalized optimal parameters, including a resolution of 0.67 mm × 0.67 mm × 1.34 mm, a flip angle of 9°, echo times of 11 ms and 20 ms, and an acquisition aligned with the Anterior Commissure - Posterior Commissure line. By integrating the SNR of magnitude images with the contrast of QSM, tSWI delivers images that stand as the most effective approach for STS visualization [62].

Additionally, an innovative fast-imaging technique combining Echo Planar Imaging (EPI) with SWI has successfully shifted STS detection from expensive, scarce, and time-consuming high-field MRI to the widely accessible 1.5 T magnetic field. This method achieved an area under the curve (AUC) of 0.98 for STS detection, highlighting its significant clinical potential for promoting early and accessible diagnosis of PD [63].

### Neuromelanin-sensitive MRI (NM-MRI)

NM-MRI and iron-sensitive imaging exhibit a complementary relationship, jointly confirming abnormalities in the SN from the dual perspectives of NM and iron content, providing multidimensional imaging evidence for PD diagnosis. Studies indicate that the overlaying of iron and NM boundaries helps readers make a more informed decision about the presence of STS [12]. NM signals show significant correlations with multiple MRI parameters: it shows a negative correlation with the CNR of QSM (Rho = –0.60), a positive correlation with the CNR of T2WI (Rho = 0.65), and a negative correlation with T1-weighted signal [34]. By utilizing techniques such as magnetization transfer contrast, NM-MRI produces high signal intensity in T1-weighted images within NM-rich regions, enabling early detection of NM loss [23].

Reduced volume or contrast of NM in the SN is a common finding in NM-MRI studies of PD patients. Studies have demonstrated that the NM distribution visualized by NM-MRI corresponds to the dopaminergic territory identified on PET imaging, and its signal intensity correlates with dopamine release capacity and disease severity in PD [64, 65]. However, studies have indicated that this technique achieves only 60–80% sensitivity in distinguishing PD patients from HC, performing no better than DAT [66]. In contrast, other research has reported a pooled sensitivity of 89% (95% confidence interval: 86%–92%) and a pooled specificity of 83% (95% CI: 76%–88%) for NM-MRI in diagnosing PD[67]. Therefore, the diagnostic value of NM-MRI in PD, as well as the diagnostic utility of the STS within NM-MRI, still require further exploration using larger sample sizes and more standardized, optimized scanning protocols.

### Ultra-high-field strengths of 7-T MRI and above

**Fig. 7:**
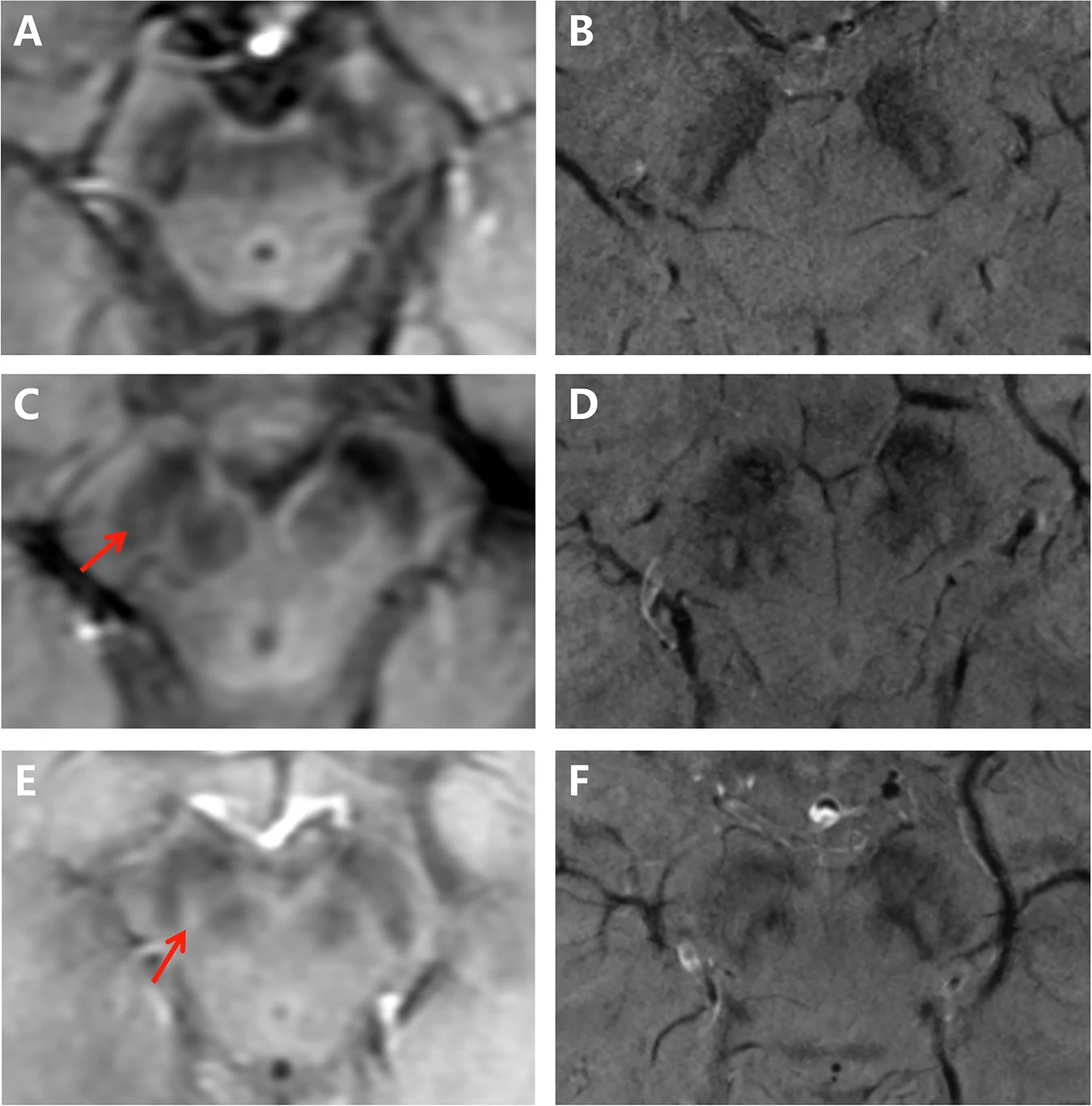
Comparison of the STS visualization between 3-T and 7-T SWI. The six panels in this figure show SWI images from three healthy subjects, with each paired image (**A**–**F**) acquired at the same anatomical level using two MRI systems with different magnetic field strengths. **A**, **C**, **E** Obtained on a 3-T scanner (Discovery 750, GE Healthcare), whereas (**B**, **D**, **F**) were obtained on a 7-T scanner (MAGNETOM Terra, Siemens Healthcare). **A**, **B** Images from a 69-year-old healthy female subject. Bilateral STS is clearly visualized on both 3-T and 7-T SWI images, with greater conspicuity on 7-T SWI. **C**, **D** Images from a 64-year-old healthy male subject. On 3-T SWI (**C**), the right-sided STS is faintly visible (red arrow), whereas the left-sided STS is not clearly identified. In contrast, bilateral STS is clearly demonstrated on 7-T SWI (**D**). **E**, **F** Images from a 52-year-old healthy female subject. On 3-T SWI (**E**), the right-sided STS is suspected but cannot be confidently identified (red arrow), and the left-sided STS is not clearly visualized. In contrast, bilateral STS is more conspicuously visualized on 7-T SWI (**F**). All the images in this figure were obtained from the Department of Radiology, First Medical Center of Chinese PLA General Hospital

Imaging of the N1 was first introduced at a field strength of 7 T, subsequently demonstrated at a field strength of 3 T, and came to be known as the “STS” [13, 27]. Ultra-high field MRI demonstrates significant advantages in the visualization and assessment of STS, due to its superior spatial resolution and SNR [68, 69] (Fig. 7). To standardize and improve the reproducibility of N1 assessment, a study based on 7 T has developed a probabilistic atlas of the N1 region corresponding to the STS, precisely pinpointing the most probable locations of STS [70]. Furthermore, leveraging the advantages of 7 T above, studies have shown that it not only enables clear visualization of the N1 structure [27, 68], but also allows distinct identification of all five nigrosomes [44], which may establish a crucial foundation for future studies aimed at characterizing even finer substructures.

In the diagnosis of PD, 7-T MRI demonstrates excellent performance in detecting the absence of the STS. Compared to the 3-T SWAN sequence, 7-T SWI achieves higher diagnostic sensitivity (7 T 93% vs 3 T 79%), specificity (7 T 100% vs 3 T 94%), and accuracy (7 T 96% vs 3 T 86%) for STS loss [71]. A meta-analysis indicates that combining 3 T and 7 T data yields a sensitivity of 97.7% and a specificity of 94.6% for diagnosing PD with STS [40]. In idiopathic RBD patients, STS loss detected on 3-T MRI increases from 63% to 87% on 7-T MRI, consistent with DAT reduction, indicating the higher potential value of 7 T in screening during the prodromal stage of the disease [72].

**Fig. 8:**
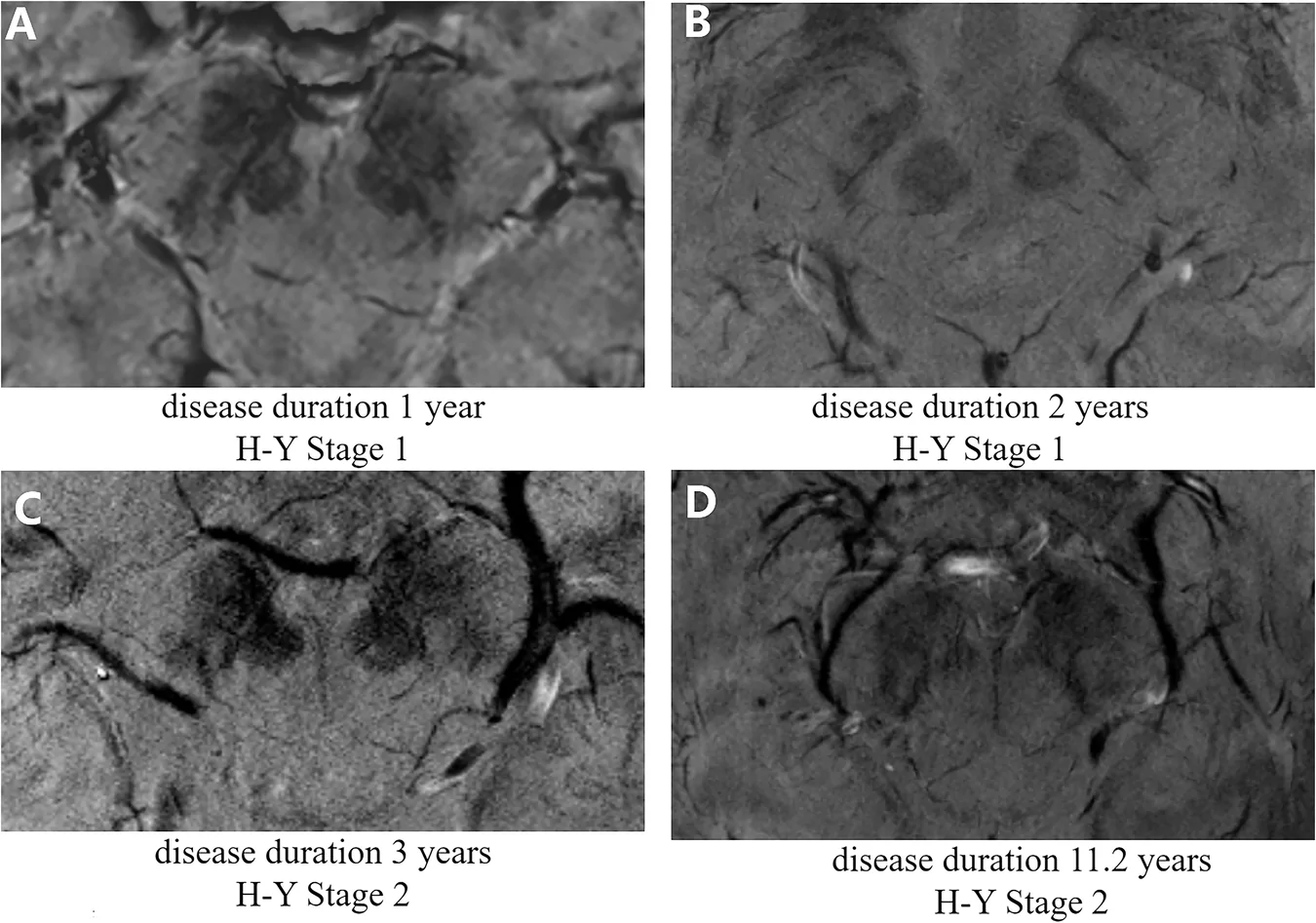
This figure shows 7-T SWI images (MAGNETOM Terra, Siemens Healthcare) from patients with early-stage PD, in which the STS is visible bilaterally in all four cases. **A** Image in a 66-year-old male patient with a disease duration of 1 year and Hoehn-Yahr (H-Y) stage 1. **B** Image in a 65-year-old female patient with disease duration of 2 years and H-Y stage 1. **C** Image in a 67-year-old female patient with a disease duration of 3 years and H-Y stage 2. **D** Image in a 64-year-old female patient with disease duration of 11.2 years and H-Y stage 2. All the images in this figure were obtained from the Department of Radiology, First Medical Center of Chinese PLA General Hospital

Furthermore, ultra-high-field imaging can better reveal the complexity of the STS. A study using 7-T SWI has shown that the STS is not necessarily present in HCs and has a false-positive rate of approximately 19% [26]. In early PD, a considerable proportion also exhibited a detectable STS (20.4% bilaterally and 34.3% unilaterally), suggesting that the presence or absence of the STS may not serve as a definitive indicator of dorsolateral SN impairment [73] (Fig. 8).

Furthermore, a large-sample 7 T study provided evidence for differential diagnosis of parkinsonism. The study indicates that, similar to PD, the majority of patients with MSA-P and PSP show absence of STS, while STS is preserved in 79.5% of those with MSA-C, which suggests that STS can help distinguish MSA subtypes. The study also revealed disease-specific spatial patterns of iron deposition: PD and PSP showed iron accumulation predominantly in the dorsal and posterior regions of the SN, MSA-P in the intermediate and posterior regions, and MSA-C in the ventral region [73]. Additionally, in CBD, another form of parkinsonism, the nigrosome structure appears normal [48].

In terms of disease progression assessment, 7-T MRI techniques—including R2* mapping, QSM, and NM imaging—have revealed correlations in PD patients between N1 abnormalities such as reduced NM signal and increased iron deposition and the severity of their motor symptoms [74]. This supports the idea that 7-T MRI can be employed to assess disease progression at the individual level [31]. Moreover, decreased 7-T T2WI signal in the N1 region correlates positively with disease severity, further validating its value in reflecting pathological progression [44].

From a technical standpoint, imaging parameter selection is significantly influenced by field strength. Higher field strengths permit shorter echo times and repetition times, providing better SNR and lower specific absorption rate compared to conventional spin-echo sequences, which makes techniques like SWI more efficient and safer at 7 T [29]. Moreover, exploration of even higher field strengths continues. A postmortem study using 9.4-T MRI did not identify significant differences in nigrosome volume or signal intensity in PD patients [75]. Another study using > 11.7-T MRI successfully visualized N1 in control samples but not in PD samples [35]. However, due to the limited sample sizes, the diagnostic value of such ultra-high-field findings requires further validation.

### ML

Traditional evaluation of STS relies on visual assessment by radiologists, a process that is inherently subjective, time-consuming, and highly dependent on examiner experience. In recent years, with the advancement of AI technologies, ML-based methods for the automatic, rapid, and accurate segmentation and identification of STS have been extensively developed.

To address the lengthy processing times associated with traditional QSM reconstruction algorithms, a study has employed a deep learning network to replace conventional iterative algorithms, which has developed an efficient and rapid susceptibility imaging processing pipeline for visualizing N1 on conventional clinical 3-T MRI [76]. In the image analysis domain, models like NINet have demonstrated the ability to efficiently and accurately identify STS from multi-sequence 3-T MRI data, achieving solid diagnostic performance (AUC = 0.87) [77]. Moreover, Ariz et al proposed a fully automated atlas-based segmentation method for the N1 region. This diagnostic model achieved a high differential diagnostic rate (AUC = 0.955) and proved valuable even in advanced PD patients where the STS is completely absent [78]. Beyond simple identification and segmentation, ML can also extract quantitative imaging features that are imperceptible to human eyes. One study utilized an ML model to quantitatively analyze the relative content of NM and iron within the N1 region, effectively distinguishing idiopathic PD from HCs. This approach overcomes the subjectivity inherent in purely visual assessments, serving as a valuable complementary tool to STS visual evaluation [78, 79].

While these ML applications demonstrate significant promise, several critical steps are essential to establish their clinical credibility and generalizability before widespread adoption. First, these models require rigorous external validation on large, multi-center cohorts. Currently, most studies are based on data from a single center or a single MRI vendor or protocol, which raises concerns about model robustness and generalizability, highlighting the need for further validation across diverse scanners, acquisition protocols, and field strengths from different institutions, as well as larger sample sizes, to enhance their reliability and broader applicability. Second, to demonstrate incremental value, the focal point of research should shift from simply comparing model performance to that of radiologists, towards investigating how ML models can augment diagnostic accuracy and efficiency. Quantifying this tangible clinical utility, especially for less experienced readers, is essential for establishing their impact.

In summary, ML has permeated all stages of STS research, from image reconstruction to disease diagnosis, substantially reducing manual effort and improving efficiency. However, transitioning these promising tools from the research domain to clinical practice hinges on future work that rigorously addresses their generalizability, robustness, and added value in multi-center settings.

### Integrated STS with other imaging biomarkers

Although STS alone is a highly specific marker for diagnosing PD, its diagnostic capability as a single indicator remains limited in clinical practice, particularly in early-stage or atypical cases [80]. Multiple studies have demonstrated that combining STS disappearance with other biomarkers, such as the content and volume of NM, deep gray matter iron content, and the volume and iron content of SNc, significantly enhances diagnostic accuracy for PD compared to using STS alone [20, 81–83]. Diagnostic precision in both prodromal and early-stage PD also markedly improves, with AUC values increasing from 0.891 to 0.983 [20]. Furthermore, literature reports indicate that combining STS assessment with NM MRI in differential diagnosis significantly enhances the accuracy of differentiating early PD from ET (AUC = 0.935, specificity 92%) [84]. Similarly, combined evaluation of STS and putaminal hypointensity (AUC = 0.93) aids in identifying MSA, as MSA patients often exhibit both STS disappearance and prominent putaminal hypointensity—a pattern not typically observed in PD [85].

In summary, integrating STS, a valuable morphological biomarker, with other imaging biomarkers such as the content of NM and iron represents the current frontier in PD imaging diagnostics. This multi-biomarker integration not only improves diagnostic efficacy but also lays the foundation for developing automated and standardized diagnostic workflows, which hold great promise to become an indispensable auxiliary tool in future clinical practice.

Based on current evidence, several technical and methodological factors can substantially influence the visualization and diagnostic performance of the STS. To provide practical guidance for clinical implementation and future research, Table 1 summarizes practical recommendations for optimizing STS acquisition, visualization, and interpretation.

**Table 1:** Practical recommendations for optimizing the STS acquisition, visualization, and interpretation

Category	Practical recommendation	Rationale	Impact on diagnostic performance
Imaging sequence	Use SWI rather than conventional T2*WI or FLAIR [29–31].	Higher CNR and susceptibility contrast improve visualization of N1and STS anatomy.	Improved image clarity and STS detection sensitivity.
	Prefer multi-echo over single-echo acquisitions [34].	Improves CNR and SNR.	Improved image quality and diagnostic confidence.
	Consider SMWI [17].	Combines SWI and QSM information, provides higher CNR, enhances venous and iron-related contrast, and reduces partial-volume effects.	Higher sensitivity and specificity for N1 detection.
	Use optimized tSWI protocols when available [58].	Integrates SNR of magnitude images with the contrast of QSM.	The most effective approach for STS visualization.
Slice thickness	Use thinner slices [36].	Reduces partial-volume effects that may obscure N1.	Improved accuracy and reproducibility.
Magnetic field strength	Prefer 3 T over 1.5 T [36]; prefer 7 T over 3 T [67].	Higher field strengths provide greater SNR, stronger susceptibility contrast, and finer anatomical delineation.	Higher sensitivity, specificity, positive predictive value, and diagnostic accuracy.
Assessment method	Use a 6-point visibility scale for each hemi-mesencephalon instead of binary classification [43, 44, 70].	Preserves information regarding partial and asymmetric nigrosome impairment.	Improved diagnostic accuracy and inter-rater agreement.
	Report reader confidence levels when feasible [37].	Accounts for diagnostic certainty and cases with unclear diagnosis.	Improved diagnostic accuracy with more confident and precise interpretation.
Multimodal Imaging	Combine SWI with QSM, NM-MRI, T1WI, and other sequences for integrated analysis [76–81].	Provides complementary information on iron deposition, NM loss, the volume of SN, and the intensity of the putamen.	Improved diagnostic precision and differential diagnostic performance.

*CNR* contrast-to-noise ratio, *SWI* susceptibility-weighted imaging, T2*WI T2*-weighted image, *FLAIR* fluid attenuated inversion recovery sequence, *N1* nigrosome-1, *STS* swallow tail sign, *SNR* signal-to-noise ratio, *SMWI* susceptibility map-weighted imaging, *QSM* quantitative susceptibility mapping, *tSWI* true susceptibility-weighted imaging, *NM-MRI* neuromelanin-sensitive magnetic resonance imaging, *T1WI* T1-weighted image, *SN* substantia nigra

## Conclusions and future perspectives

Researchers are making significant efforts to identify non-invasive, convenient, and highly diagnostic imaging biomarkers for neurodegenerative diseases such as PD. In this review, we clearly and comprehensively elucidate the anatomical and pathophysiological mechanisms underlying the appearance and disappearance of STS. Furthermore, the majority of the presented literature demonstrated that STS, as an adjunctive tool, exhibits high sensitivity and specificity in distinguishing PD from HCs, and shows potential in differentiating various forms of parkinsonism. However, some studies have questioned its diagnostic value. To evaluate its diagnostic utility more definitively, future research should incorporate larger sample sizes and conduct long-term follow-up studies. Moreover, because STS is a qualitative imaging marker based on signal intensity, its visibility can be influenced by variations in scanning protocols, including magnetic field strength and sequence type. Consequently, developing optimized and standardized scanning protocols is essential to achieve clearer visualization of the STS. Meanwhile, quantitative MRI techniques such as QSM provide a complementary strategy by directly quantifying magnetic susceptibility changes. Notably, advancements in data post-processing, such as Apart-QSM and χ-seperation, enable the separation of paramagnetic and diamagnetic substances, improving the accuracy of iron deposition assessment [86–88]. Integrating qualitative and quantitative biomarkers within a multimodal imaging framework may enhance diagnostic accuracy and offer deeper insights into the pathophysiological mechanisms underlying PD. Furthermore, large-scale sample cohorts, standardized imaging protocols, and the adoption of quantitative techniques will also help establish a foundation for building big data environments. With technological advancements, the use of super-resolution reconstruction to enhance 3-T images to a quality comparable to that of 7-T images is a promising future research direction for the identification of STS, as it could reduce hardware requirements and save costs.

## Data Availability

Not applicable.

## Abbreviations

AP: Atypical parkinsonian syndrome
AUC: Area under the curve
CBD: Corticobasal degeneration
CNR: Contrast-to-noise ratio
ET: Essential tremor
HC: Healthy control
ML: Machine learning
MRI: Magnetic resonance imaging
MSA: Multiple system atrophy
MSA-C: MSA cerebellar variant
MSA-P: MSA parkinsonian variant
N1: Nigrosome-1
N5: Nigrosome-5
NM: Neuromelanin
NM-MRI: Neuromelanin-sensitive MRI
PD: Parkinson’s disease
PET: Positron emission tomography
PSP: Progressive supranuclear palsy
QSM: Quantitative susceptibility mapping
RBD: Rapid eye movement sleep behavior disorder
SMWI: Susceptibility map-weighted imaging
SN: Substantia nigra
SNc: Substantia nigra pars compacta
SNR: Signal-to-noise ratio
SNr: Substantia nigra pars reticulata
SPECT: Single-photon emission computed tomography
STS: Swallow tail sign
SWI: Susceptibility-weighted imaging
T2-FLAIR: T2-weighted fluid-attenuated inversion recovery sequence
T2WI: T2-weighted image
tSWI: True susceptibility-weighted imaging

## References

[1] Bloem BR, Okun MS, Klein C (2021) Parkinson’s disease. Lancet 397:2284–2303 10.1016/S0140-6736(21)00218-X 33848468

[2] Maheshwari S, Akram H, Bulstrode H et al (2024) Dopaminergic cell replacement for Parkinson’s disease: addressing the intracranial delivery hurdle. J Parkinsons Dis 14:415–435 10.3233/JPD-230328 PMC11091588 38457149

[3] Su D, Cui Y, He C et al (2025) Projections for prevalence of Parkinson’s disease and its driving factors in 195 countries and territories to 2050: modelling study of Global Burden of Disease Study 2021. BMJ 388:e080952 10.1136/bmj-2024-080952 PMC11881235 40044233

[4] Postuma RB, Berg D, Stern M et al (2015) MDS clinical diagnostic criteria for Parkinson’s disease. Mov Disord 30:1591–1601 10.1002/mds.26424 26474316

[5] Stezin A, Naduthota RM, Botta R et al (2018) Clinical utility of visualisation of nigrosome-1 in patients with Parkinson’s disease. Eur Radiol 28:718–726 10.1007/s00330-017-4950-5 28779393

[6] Li W, Lao-Kaim NP, Roussakis AA et al (2018) 11 C-PE2I and 18 F-dopa PET for assessing progression rate in Parkinson’s: a longitudinal study. Mov Disord 33:117–127 10.1002/mds.27183 29082547

[7] Kaasinen V, Vahlberg T (2017) Striatal dopamine in parkinson disease: a meta-analysis of imaging studies. Ann Neurol 82:873–882 10.1002/ana.25103 29165839

[8] Meyer PT, Frings L, Rücker G, Hellwig S (2017) ^18^F-FDG PET in Parkinsonism: differential diagnosis and evaluation of cognitive impairment. J Nucl Med 58:1888–1898 10.2967/jnumed.116.186403 28912150

[9] Walker Z, Gandolfo F, Orini S et al (2018) Clinical utility of FDG PET in Parkinson’s disease and atypical parkinsonism associated with dementia. Eur J Nucl Med Mol Imaging 45:1534–1545 10.1007/s00259-018-4031-2 PMC6061481 29779045

[10] Matsuoka K, Ono M, Takado Y et al (2022) High-contrast imaging of α-synuclein pathologies in living patients with multiple system atrophy. Mov Disord 37:2159–2161 10.1002/mds.29186 PMC9804399 36041211

[11] Xiang J, Tao Y, Xia Y et al (2023) Development of an α-synuclein positron emission tomography tracer for imaging synucleinopathies. Cell 186:3350–3367.e19 10.1016/j.cell.2023.06.004 PMC10527432 37421950

[12] Haller S, He N, Schwarz S, Haacke EM (2025) Clinical MRI biomarkers to differentiate parkinson disease from its mimics. Radiology 317:e242044 10.1148/radiol.242044 41085393

[13] Schwarz ST, Afzal M, Morgan PS, Bajaj N, Gowland PA, Auer DP (2014) The ‘swallow tail’ appearance of the healthy nigrosome—a new accurate test of Parkinson’s disease: a case-control and retrospective cross-sectional MRI study at 3T. PLoS One 9:e93814 10.1371/journal.pone.0093814 PMC3977922 24710392

[14] Lee J-Y, Martin-Bastida A, Murueta-Goyena A et al (2022) Multimodal brain and retinal imaging of dopaminergic degeneration in parkinson disease. Nat Rev Neurol 18:203–220 10.1038/s41582-022-00618-9 35177849

[15] Damier P, Hirsch EC, Agid Y, Graybiel AM (1999) The substantia nigra of the human brain. I. Nigrosomes and the nigral matrix, a compartmental organization based on calbindin D(28K) immunohistochemistry. Brain 122:1421–1436 10.1093/brain/122.8.1421 10430829

[16] Damier P, Hirsch EC, Agid Y, Graybiel AM (1999) The substantia nigra of the human brain. II. Patterns of loss of dopamine-containing neurons in Parkinson's disease. Brain 122:1437–1448. 10.1093/brain/122.8.1437 10430830

[17] Kim EY, Sung YH, Lee J (2019) Nigrosome 1 imaging: technical considerations and clinical applications. Br J Radiol 92:20180842 10.1259/bjr.20180842 PMC6732928 31067082

[18] Reiter E, Mueller C, Pinter B et al (2015) Dorsolateral nigral hyperintensity on 3.0T susceptibility-weighted imaging in neurodegenerative parkinsonism. Mov Disord 30:1068–1076 10.1002/mds.26171 25773707

[19] Brammerloh M, Kirilina E, Alkemade A et al (2022) Swallow tail sign: revisited. Radiology 305:674–677 10.1148/radiol.212696 35972361

[20] He N, Ghassaban K, Huang P et al (2021) Imaging iron and neuromelanin simultaneously using a single 3D gradient echo magnetization transfer sequence: combining neuromelanin, iron and the nigrosome-1 sign as complementary imaging biomarkers in early stage Parkinson’s disease. Neuroimage 230:117810 10.1016/j.neuroimage.2021.117810 33524572

[21] Takahashi H, Watanabe Y, Tanaka H et al (2018) Quantifying changes in nigrosomes using quantitative susceptibility mapping and neuromelanin imaging for the diagnosis of early-stage Parkinson’s disease. Br J Radiol 91:20180037 10.1259/bjr.20180037 PMC6223299 29498540

[22] Takahashi H, Watanabe Y, Tanaka H et al (2018) Comprehensive MRI quantification of the substantia nigra pars compacta in Parkinson’s disease. Eur J Radiol 109:48–56 10.1016/j.ejrad.2018.06.024 30527311

[23] He N, Chen Y, LeWitt PA, Yan F, Haacke EM (2023) Application of neuromelanin MR imaging in Parkinson disease. J Magn Reson Imaging 57:337–352. 10.1002/jmri.28414 PMC10086789 36017746

[24] Sulzer D, Cassidy C, Horga G et al (2018) Neuromelanin detection by magnetic resonance imaging (MRI) and its promise as a biomarker for Parkinson’s disease. NPJ Parkinsons Dis 4:11 10.1038/s41531-018-0047-3 PMC5893576 29644335

[25] Kau T, Hametner S, Endmayr V et al (2019) Microvessels may confound the “Swallow Tail Sign” in normal aged midbrains: a postmortem 7 T SW-MRI study. J Neuroimaging 29:65–69 10.1111/jon.12576 30407679

[26] Schmidt MA, Engelhorn T, Marxreiter F et al (2017) Ultra high-field SWI of the substantia nigra at 7T: reliability and consistency of the swallow-tail sign. BMC Neurol 17:194 10.1186/s12883-017-0975-2 PMC5658950 29073886

[27] Blazejewska AI, Schwarz ST, Pitiot A et al (2013) Visualization of nigrosome 1 and its loss in PD: pathoanatomical correlation and in vivo 7 T MRI. Neurology 81:534–540 10.1212/WNL.0b013e31829e6fd2 PMC3775686 23843466

[28] Biesemeier A, Eibl O, Eswara S et al (2016) Elemental mapping of neuromelanin organelles of human substantia nigra: correlative ultrastructural and chemical analysis by analytical transmission electron microscopy and nano-secondary ion mass spectrometry. J Neurochem 138:339–353 10.1111/jnc.13648 27121280

[29] Haller S, Haacke EM, Thurnher MM, Barkhof F (2021) Susceptibility-weighted imaging: technical essentials and clinical neurologic applications. Radiology 299:3–26 10.1148/radiol.2021203071 33620291

[30] Sotoudeh H, Sarrami AH, Wang JX et al (2021) Susceptibility-weighted imaging in neurodegenerative disorders: a review. J Neuroimaging 31:459–470 10.1111/jon.12841 33624404

[31] Madelung CF, Løkkegaard A, Fuglsang SA et al (2025) High-resolution mapping of substantia nigra in Parkinson’s disease using 7 tesla magnetic resonance imaging. NPJ Parkinsons Dis 11:113 10.1038/s41531-025-00972-7 PMC12056087 40328786

[32] Gorell JM, Ordidge RJ, Brown GG et al (1995) Increased iron-related MRI contrast in the substantia nigra in Parkinson’s disease. Neurology 45:1138–1143 10.1212/wnl.45.6.1138 7783878

[33] Oh SW, Shin NY, Lee JJ et al (2016) Correlation of 3D FLAIR and dopamine transporter imaging in patients with parkinsonism. AJR Am J Roentgenol 207:1089–1094 10.2214/AJR.16.16092 27489952

[34] Lee T-W, Chen CY, Chen K et al (2021) Evaluation of the swallow-tail sign and correlations of neuromelanin signal with susceptibility and relaxations. Tomography 7:107–119 10.3390/tomography7020010 PMC8103261 33801685

[35] Meijer FJ, Steens SC, van Rumund A et al (2016) Nigrosome-1 on susceptibility weighted imaging to differentiate Parkinson’s disease from atypical parkinsonism: an in vivo and ex vivo pilot study. Pol J Radiol 81:363–369 10.12659/PJR.897090 PMC4975367 27559425

[36] Shams S, Fällmar D, Schwarz S et al (2017) MRI of the swallow tail sign: A useful marker in the diagnosis of Lewy body dementia? AJNR Am J Neuroradiol 38:1737–1741 10.3174/ajnr.A5274 PMC7963704 28705819

[37] Xing Y, Naidu S, Martín-Bastida A et al (2025) Diagnostic value of swallow tail sign at brain MRI in patients with clinically uncertain parkinsonian syndrome. Radiology 316:e240680 10.1148/radiol.240680 40626875

[38] Michler E, Kaiser D, Eleftheriadou K, Falkenburger B, Kotzerke J, Hoberück S (2021) Comparison of 6-[(18)F]FDOPA PET with Nigrosome 1 detection in patients with parkinsonism. EJNMMI Res 11:16 10.1186/s13550-021-00758-x PMC7884547 33590381

[39] Tseriotis VS, Eleftheriadou K, Mavridis T, Konstantis G, Falkenburger B, Arnaoutoglou M (2025) Is the swallow tail sign a useful imaging biomarker in clinical neurology? A systematic review. Mov Disord Clin Pr 12:134–147 10.1002/mdc3.14304 PMC11802665 39688317

[40] Mahlknecht P, Krismer F, Poewe W, Seppi K (2017) Meta-analysis of dorsolateral nigral hyperintensity on magnetic resonance imaging as a marker for Parkinson’s disease. Mov Disord 32:619–623 10.1002/mds.26932 28151553

[41] Barber TR, Griffanti L, Bradley KM et al (2020) Nigrosome 1 imaging in REM sleep behavior disorder and its association with dopaminergic decline. Ann Clin Transl Neurol 7:26–35 10.1002/acn3.50962 PMC6952317 31820587

[42] Zhang X, Chai C, Ghassaban K et al (2021) Assessing brain iron and volume of subcortical nuclei in idiopathic rapid eye movement sleep behavior disorder. Sleep 44:zsab131 10.1093/sleep/zsab131 34015127

[43] Prasuhn J, Neumann A, Strautz R et al (2021) Clinical MR imaging in Parkinson’s disease: How useful is the swallow tail sign? Brain Behav 11:e02202 10.1002/brb3.2202 PMC8323030 34032020

[44] Schwarz ST, Mougin O, Xing Y et al (2018) Parkinson’s disease related signal change in the nigrosomes 1–5 and the substantia nigra using T2* weighted 7T MRI. Neuroimage Clin 19:683–689 10.1016/j.nicl.2018.05.027 PMC5986169 29872633

[45] Kim DS, Tung GA, Akbar U, Friedman JH (2021) The evaluation of the swallow tail sign in patients with parkinsonism and gait disorders. J Neurol Sci 428:117581 10.1016/j.jns.2021.117581 34333378

[46] Bae YJ, Kim JM, Kim E et al (2016) Loss of nigral hyperintensity on 3 Tesla MRI of Parkinsonism: comparison with (123) I-FP-CIT SPECT. Mov Disord 31:684–692 10.1002/mds.26584 26990970

[47] Oustwani CS, Korutz AW, Lester MS, Kianirad Y, Simuni T, Hijaz TA (2017) Can loss of the swallow tail sign help distinguish between Parkinson Disease and the Parkinson-Plus syndromes? Clin Imaging 44:66–69 10.1016/j.clinimag.2017.04.005 28460362

[48] Frosini D, Ceravolo R, Tosetti M, Bonuccelli U, Cosottini M (2016) Nigral involvement in atypical parkinsonisms: evidence from a pilot study with ultra-high field MRI. J Neural Transm (Vienna) 123:509–513 10.1007/s00702-016-1529-2 26943945

[49] Whitwell JL, Höglinger GU, Antonini A et al (2017) Radiological biomarkers for diagnosis in PSP: Where are we and where do we need to be? Mov Disord 32:955–971 10.1002/mds.27038 PMC5511762 28500751

[50] Rizzo G, De Blasi R, Capozzo R et al (2019) Loss of swallow tail sign on susceptibility-weighted imaging in dementia with Lewy bodies. J Alzheimers Dis 67:61–65 10.3233/JAD-180687 30475767

[51] Tseriotis VS, Mavridis T, Eleftheriadou K et al (2024) Loss of the “swallow tail sign” on susceptibility-weighted imaging in the diagnosis of dementia with Lewy bodies: a systematic review and meta-analysis. J Neurol 271:3754–3763 10.1007/s00415-024-12381-6 38801432

[52] Falcitano L, Calizzano F, Mattioli P et al (2026) MRI susceptibility map weighted imaging (SMWI) as a neurodegeneration biomarker in the prodromal to overt alpha-synucleinopathy continuum. J Parkinsons Dis 16:61–71 10.1177/1877718X251387027 PMC13347558 41334620

[53] Weber CE, Ebert A, Platten M, Gass A, Eisele P (2020) Susceptibility-weighted 3T MRI of the swallow tail sign in multiple sclerosis: a case control study. J Neuroimaging 30:766–768 10.1111/jon.12775 32857891

[54] Cui XL, Liu S, Zhang YM, Zhao HD, Shi JQ (2023) Loss of swallow tail sign on susceptibility-weighted imaging in neurosyphilis mimicking Alzheimer’s disease: a case report. Acta Neurol Belg 123:1581–1583 10.1007/s13760-022-02107-8 36178672

[55] Noh Y, Sung YH, Lee J, Kim EY (2015) Nigrosome 1 detection at 3T MRI for the diagnosis of early-stage idiopathic parkinson disease: assessment of diagnostic accuracy and agreement on imaging asymmetry and clinical laterality. AJNR Am J Neuroradiol 36:2010–2016 10.3174/ajnr.A4412 PMC7964859 26294646

[56] Sui WF, Zhang HY, Xu J, Xu Y, Wu JT (2016) Application of swallow tail appearance in patients with Parkinson’s disease. Zhonghua Yi Xue Za Zhi 96:30–32 10.3760/cma.j.issn.0376-2491.2016.01.007 26792604

[57] Chen M, Wang Y, Zhang C et al (2023) Free water and iron content in the substantia nigra at different stages of Parkinson’s disease. Eur J Radiol 167:111030 10.1016/j.ejrad.2023.111030 37579561

[58] Liu S, Mok K, Neelavalli J et al (2014) Improved MR venography using quantitative susceptibility-weighted imaging. J Magn Reson Imaging 40:698–708 10.1002/jmri.24413 PMC4059787 24923249

[59] Nam Y, Gho SM, Kim DH, Kim EY, Lee J (2017) Imaging of nigrosome 1 in substantia nigra at 3T using multiecho susceptibility map-weighted imaging (SMWI). J Magn Reson Imaging 46:528–536 10.1002/jmri.25553 27859983

[60] Sun J, Lai Z, Ma J et al (2020) Quantitative evaluation of iron content in idiopathic rapid eye movement sleep behavior disorder. Mov Disord 35:478–485 10.1002/mds.27929 31846123

[61] Wang N, Liu XL, Li L et al (2021) Screening for early-stage Parkinson’s disease: swallow tail sign on MRI susceptibility map-weighted images compared with PET. J Magn Reson Imaging 53:722–730 10.1002/jmri.27386 33096586

[62] Cheng Z, He N, Huang P et al (2020) Imaging the nigrosome 1 in the substantia nigra using susceptibility weighted imaging and quantitative susceptibility mapping: an application to Parkinson’s disease. Neuroimage Clin 25:102103 10.1016/j.nicl.2019.102103 PMC6933220 31869769

[63] Hamed MR, Kenawy MA, Mohamed S, El-Samahy M, Ereiba KT, Eissa A (2025) Fast susceptibility-weighted imaging using echo planar imaging at 1.5 T for swallow tail sign biomarker detection in Parkinson’s disease. Radiography (Lond) 31:102903 10.1016/j.radi.2025.102903 40020415

[64] Cassidy CM, Zucca FA, Girgis RR et al (2019) Neuromelanin-sensitive MRI as a noninvasive proxy measure of dopamine function in the human brain. Proc Natl Acad Sci USA 116:5108–5117 10.1073/pnas.1807983116 PMC6421437 30796187

[65] Schwarz ST, Xing Y, Tomar P, Bajaj N, Auer DP (2017) In vivo assessment of brainstem depigmentation in parkinson disease: potential as a severity marker for multicenter studies. Radiology 283:789–798 10.1148/radiol.2016160662 27820685

[66] Fabbri M, Reimão S, Carvalho M et al (2017) Substantia nigra neuromelanin as an imaging biomarker of disease progression in Parkinson’s disease. J Parkinsons Dis 7:491–501 10.3233/JPD-171135 28671143

[67] Cho SJ, Bae YJ, Kim JM et al (2021) Diagnostic performance of neuromelanin-sensitive magnetic resonance imaging for patients with Parkinson’s disease and factor analysis for its heterogeneity: a systematic review and meta-analysis. Eur Radiol 31:1268–1280 10.1007/s00330-020-07240-7 32886201

[68] Cosottini M, Frosini D, Pesaresi I et al (2014) MR imaging of the substantia nigra at 7 T enables diagnosis of Parkinson disease. Radiology 271:831–838 10.1148/radiol.14131448 24601752

[69] Wang X, Xiong Y, Duan C et al (2025) The disease-specific structural pattern in Parkinson’s disease and its cortical characteristics associated with gene function: a 7-tesla MRI study. J Neurol 272:300 10.1007/s00415-025-13035-x 40159562

[70] Lancione M, Donatelli G, Migaleddu G et al (2025) High resolution multi-parametric probabilistic in vivo atlas of dorsolateral nigral hyperintensity via 7 T MRI. Sci Data 12:958 10.1038/s41597-025-05325-w PMC12145438 40483299

[71] Cosottini M, Frosini D, Pesaresi I et al (2015) Comparison of 3T and 7T susceptibility-weighted angiography of the substantia nigra in diagnosing parkinson disease. AJNR Am J Neuroradiol 36:461–466 10.3174/ajnr.A4158 PMC8013050 25376811

[72] Frosini D, Cosottini M, Donatelli G et al (2017) Seven tesla MRI of the substantia nigra in patients with rapid eye movement sleep behavior disorder. Parkinsonism Relat Disord 43:105–109 10.1016/j.parkreldis.2017.08.002 28781201

[73] Su D, Zhang Z, Zhang Z et al (2025) Dorsal hyperintensity and iron deposition patterns in the substantia nigra of Parkinson’s disease, idiopathic REM sleep behavior disorder, and parkinson-plus syndromes at 7T MRI: a prospective diagnostic study. Transl Neurodegener 14:35 10.1186/s40035-025-00495-4 PMC12231697 40616129

[74] Wang X, Xiong Y, Hu J et al (2025) Thalamic functional connectivity on 7-tesla magnetic resonance imaging and its relation to motor signs in early-stage Parkinson’s disease. Quant Imaging Med Surg 15:623–635 10.21037/qims-24-1498 PMC11744139 39839040

[75] Massey LA, Miranda MA, Al-Helli O et al (2017) 9.4 T MR microscopy of the substantia nigra with pathological validation in controls and disease. Neuroimage Clin 13:154–163 10.1016/j.nicl.2016.11.015 PMC5144755 27981030

[76] Jo M, Oh SH (2020) A preliminary attempt to visualize nigrosome 1 in the substantia nigra for Parkinson’s disease at 3T: an efficient susceptibility map-weighted imaging (SMWI) with quantitative susceptibility mapping using deep neural network (QSMnet). Med Phys 47:1151–1160 10.1002/mp.13999 31883389

[77] Wang C, He N, Zhang Y et al (2024) Enhancing nigrosome-1 sign identification via interpretable AI using true susceptibility weighted imaging. J Magn Reson Imaging 60:1904–1915 10.1002/jmri.29245 38236577

[78] Ariz M, Martínez M, Alvarez I et al (2024) Automatic segmentation and quantification of Nigrosome-1 neuromelanin and iron in MRI: a candidate biomarker for Parkinson’s disease. J Magn Reson Imaging 60:534–547 10.1002/jmri.29073 37915245

[79] Cheng Z, Zhang J, He N et al (2019) Radiomic features of the Nigrosome-1 region of the substantia nigra: using quantitative susceptibility mapping to assist the diagnosis of idiopathic Parkinson’s disease. Front Aging Neurosci 11:167 10.3389/fnagi.2019.00167 PMC6648885 31379555

[80] Chau MT, Todd G, Wilcox R, Agzarian M, Bezak E (2020) Diagnostic accuracy of the appearance of Nigrosome-1 on magnetic resonance imaging in Parkinson’s disease: a systematic review and meta-analysis. Parkinsonism Relat Disord 78:12–20 10.1016/j.parkreldis.2020.07.002 32668370

[81] Fushimi Y, Ghassaban K, Sethi SK et al (2026) Integrated evaluation of Nigrosome 1 sign, neuromelanin-sensitive MR and iron deposition. Jpn J Radio 44:24–34 10.1007/s11604-025-01858-7 PMC12769993 40824349

[82] Jokar M, Jin Z, Huang P et al (2023) Diagnosing Parkinson’s disease by combining neuromelanin and iron imaging features using an automated midbrain template approach. Neuroimage 266:119814 10.1016/j.neuroimage.2022.119814 36528314

[83] Cao Q, Han X, Tang D et al (2023) Diagnostic value of combined magnetic resonance imaging techniques in the evaluation of Parkinson disease. Quant Imaging Med Surg 13:6503–6516 10.21037/qims-23-87 PMC10585559 37869346

[84] Jin L, Wang J, Wang C et al (2019) Combined visualization of nigrosome-1 and neuromelanin in the substantia nigra using 3T MRI for the differential diagnosis of essential tremor and de novo Parkinson’s disease. Front Neurol 10:100 10.3389/fneur.2019.00100 PMC6379476 30809189

[85] Wang N, Yang H, Li C, Fan G, Luo X (2017) Using ‘swallow-tail’ sign and putaminal hypointensity as biomarkers to distinguish multiple system atrophy from idiopathic Parkinson’s disease: a susceptibility-weighted imaging study. Eur Radiol 27:3174–3180 10.1007/s00330-017-4743-x 28105503

[86] Dong L, Zhou W, An R et al (2025) Paramagnetic susceptibility mapping better quantifies brain iron content in Parkinson’s disease with RBD. NPJ Parkinsons Dis 11:192 10.1038/s41531-025-01043-7 PMC12217153 40595757

[87] Li Z, Feng R, Liu Q et al (2023) APART-QSM: an improved sub-voxel quantitative susceptibility mapping for susceptibility source separation using an iterative data fitting method. NeuroImage 274:120148 10.1016/j.neuroimage.2023.120148 37127191

[88] Kim J, Kim M, Ji S et al (2025) In-vivo high-resolution χ-separation at 7T. NeuroImage 308:121060 10.1016/j.neuroimage.2025.121060 39884410

